# Measuring the Effect of Pack Shape on Gravel’s Pore Characteristics and Permeability Using X-ray Diffraction Computed Tomography

**DOI:** 10.3390/ma15176173

**Published:** 2022-09-05

**Authors:** Jiayi Peng, Zhenzhong Shen, Jiafa Zhang

**Affiliations:** 1 State Key Laboratory of Hydrology-Water Resources and Hydraulic Engineering, Hohai University, Nanjing 210098, China; 2 National Engineering Research Center for Dam Safety, Changjiang River Scientific Research Institute, Wuhan 430010, China

**Keywords:** pack shape, gravel, X-ray computed tomography, image segmentation, surface area measurement, pore characteristic, permeability characteristic

## Abstract

Particle shape is one of the critical parameter factors that affect gravel’s pore structure and permeability. However, few studies have considered its effects on engineering applications due to the difficulty of conducting laboratory tests. To overcome these difficulties, new methods of estimating the gravel pack shape that involve manual work and measuring the surface area of particles and pores based on support vector machine segmentation and the reconstruction of X-ray diffraction computed tomography (CT) images were proposed. Under the same conditions, CT tests were carried out on gravel packs and two other regular-shaped particle packs to investigate the influence of particle shape on the fractal dimension of gravel’s pore–particle interface and the specific surface area of the pore network. Additionally, permeability tests were performed to study the effect of particle shape on gravel’s hydraulic conductivity. The results showed that a gravel pack with a larger aspect ratio and a smaller roundness had a larger specific pore network surface area and a more complex pore structure, leading to lower permeability. This kind of gravel had a more significant length, quantity, and tortuosity of the seepage path when seepage occurred in a two-dimensional seepage field simulation. Therefore, we suggest that the filter materials of hydraulic projects should preferably use blasting gravel with a larger aspect ratio and smaller roundness to achieve better anti-seepage properties. In addition, projects can increase pores’ specific surface area using our method as a control factor in filter construction.

## 1. Introduction

Permeability is one of the critical parameters of concern in hydraulic engineering, as seepage is the leading cause of earth rock dam failure, accounting for approximately 25% of such failures worldwide [1]. Gravel is used as the filter material for earth rock-fill dams to ensure seepage stability. According to the specifications for earth rock dams in the United States and China, the particle size distribution and dry density (or porosity) are the main construction control factors in filter design. Due to the influence of field test conditions, the design and construction of water conservancy projects require a formula to accurately predict the permeability of gravel based on the particle size distribution, dry density, and other parameters that can be obtained conveniently from the project site. Particle shape is an essential factor that affects the pore structure and permeability of gravel [2]. The hydraulic conductivity of natural gravel is more than five times that of blasted gravel under the same conditions due to their different particle shapes [3]. However, there are few studies on the influence of blasting gravel shape on its permeability in the context of engineering applications.

Many classic formulas [4,5,6,7] and empirical formulas [8,9,10] were proposed to predict saturated soil’s permeability based on geometric parameters related to pore characteristics, among which the Kozeny–Carman (KC) formula [6] is the most accurate [11,12]. However, before using the KC formula, it is necessary to modify it using the available engineering parameters and to measure the surface area through laboratory tests. The surface area is one of the main characterization parameters of pores and it varies with particle shape, surface characteristics, and size distribution. Therefore, it is not easy to use the KC formula in practical water conservancy projects due to the challenges involved in obtaining the calculation parameters.

Recent studies on a particle shape’s influence on gravel’s pore structure and permeability were mainly conducted through simulations [13,14,15,16,17,18,19,20,21]. Most of them used a program to build a virtual particle pack and calculate the seepage field because the shape factor of each particle, pore distribution, throat diameter of the seepage channel, and other parameters can easily be obtained from simulations. However, their conclusions cannot be verified with tests since the parameters in their studies cannot be obtained through laboratory or field tests, meaning that these results are not easily applicable to engineering applications. For instance, Cote [19], Mostefa [20], and Su [21] studied different materials based on simulations and concluded that the influence of particle shape on the permeability and pores was negligible, which is contrary to the engineering data [3,22]. Therefore, a comprehensive study of the problem must use a combination of experiments and simulations and consider laboratory and field test conditions to increase the applicability of the results to water conservancy projects. Currently, there are three problems with combining tests and pore scale simulations: (1) the internal structure of gravel does not change the accumulation of particles when it is measured in a permeability test, (2) it is difficult to describe the relationship between the microstructure and properties using the parameters commonly used in engineering, and (3) it is difficult to evaluate the shape of a gravel pack from laboratory tests.

An X-ray diffraction computer tomography (CT) test can obtain the pore structure of gravel without affecting its accumulation and is the main method of studying the characteristics of gravel from a microscopic perspective. CT involves converting the matrix of absorption coefficient arrangement into different gray-scale pixel blocks through an analog/digital converter according to the different absorption and transmittance of the rays of different density materials [23]. The primary image segmentation methods, such as threshold segmentation, region segmentation, edge segmentation, and histogram methods [24], are unsuitable for studying the gravel used in engineering because these methods require the manual adjustment of parameters for different test samples. Artificial intelligence (AI) segmentation has the advantage of self-adjusting parameters through deep learning and is suitable for engineering. Currently, AI image segmentation technology is mainly used in the field of medicine [25,26] and few relevant reports have been published on soil and rock. A series of slice images of a gravel pack can be obtained using CT to scan the pore structure of the gravel. However, it is challenging to use suitable parameters to describe the microstructure characteristics in images and to study the relationship between microscopic parameters and macroscopic properties. There are two ways to overcome these difficulties. One is to directly analyze the two-dimensional images to study the changes in the corresponding microstructure when the properties of the test samples change [27,28,29,30,31,32]. The other is to use CT images to reconstruct the three-dimensional model of the test sample and then study the relationship between the reconstructed model’s structural characteristics and the sample’s properties [33,34,35]. Because the algorithm infers the model between slices during reconstruction, the reconstructed model differs slightly from the test sample. Therefore, when studying the permeability of gravel from a microscopic perspective, it is necessary to analyze both CT images and reconstruction models. The difficulty with studying the shape of gravel from laboratory tests is that the shape factor used should have three-dimensional physical significance. The roundness, irregularity, and sphericity that describe the shape of a particle profile are all two-dimensional parameters [36,37,38,39,40,41,42,43,44]. The method of measuring the three-dimensional shape factor of gravel proposed in our previous paper [22] was suitable for this study. A tested gravel pack usually includes thousands of particles, and it is challenging to complete the measurement of the shape of each particle because of the enormous workload involved. Therefore, it is necessary to estimate the shape of the gravel pack through sampling measurement. The rationality of the sampling method design and whether there is a correlation between the shape of gravel bags estimated by sampling and the shape-related properties need to be further discussed.

In light of the above facts, this work aimed to study the influence of particle shape on the pore structure and permeability of gravel by combining laboratory tests and simulations to provide suggestions for the design and construction of filter materials in water conservancy projects and the development of permeability-predicting formulas. Therefore, X-ray CT scanning and constant head tests were conducted on gravel and two other regular-shaped particles. Meanwhile, a new method was proposed for measuring the specific surface area of the gravel pore network based on the segmentation and reconstruction of CT images using SVM to ensure that the particle accumulation was unchanged. Additionally, the relationship between the shape factor and pore characteristics of actual gravel in terms of the fractal dimension, specific surface area, and permeability is discussed. The results of this study provide suggestions for the design and construction of the earth rock dams’ filter and may help to increase the application of the predicting formula related to the shape factor and surface area of gravel in engineering.

## 2. Materials and Methods

### 2.1. Materials

**Figure 1:**
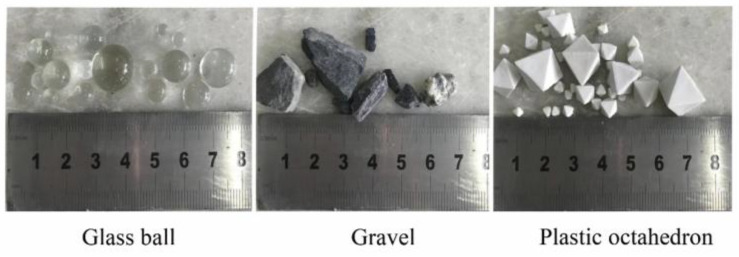
Materials used.

The experimental materials were blasted gravels from the Shuibuya Dam yard in China, which blasts limestone fragments. They were sieved and washed to remove impurities and dust before being studied. Glass balls and plastic octahedrons were selected for comparison to reduce the workload when artificially measuring the physical dimensions. Since there is no seepage deformation involved in low-head permeability tests, the different materials did not affect the test results. The sample particle sizes ranged from 2 mm to 20 mm to diminish the size effect on the test results. The materials are shown in Figure 1.

### 2.2. Experimental Process and Operation

**Figure 2:**
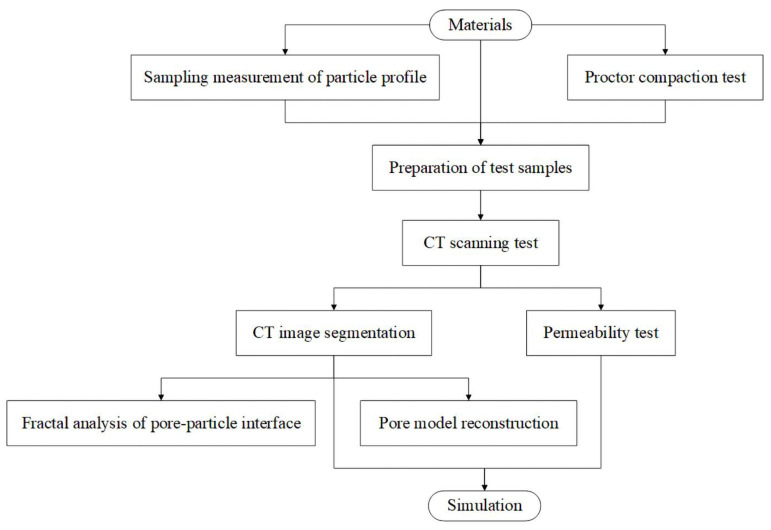
Experimental process.

Figure 2 depicts the experiment flow of this study. First, we manually sampled the gravel and measured the shape. The proctor compaction test examined the materials’ maximum and minimum dry density. Then, we designed the particle size distribution and porosity of laboratory test samples according to the particle size distribution of the sampled gravel and the dry density range. Table 1 shows nine test samples, and the shape was the only variable factor. Test samples were prepared as described in Table 1 and were used to fill a permeameter in three layers of the same thickness for homogenization. The permeameter was a plastic cylinder with an inner diameter of 10 cm. The lower water inlet chamber was fitted with a connecting pipe for the pressure measuring tube, and the top was fitted with an overflow pipe. The filling height of the sample was 7 cm, which was more than three times the maximum particle size, to eliminate the effect of size on the results. Next, the CT scanning test was conducted on the samples to obtain their pore structures, and the permeability test was carried out on the samples to study their hydraulic conductivities. We segmented the CT scanning test results to extract the samples’ pores for fractal analysis and three-dimensional model reconstruction. Finally, the permeability test was simulated two-dimensionally based on the CT images.

#### 2.2.1. Sampling Measurement of the Gravel’s Shape and the Design of the Particle Size Distribution

We defined the aspect ratio and roundness as parameters to describe the particle shape. The aspect ratio (*α*) is the length-to-width ratio, and the roundness (*S*) is defined as the ratio of the circumference of the equivalent area of the projected area to the projected contour’s actual circumference, which is as follows:

(1) $$\begin{array}{c} \varphi =\frac{I}{L}\; \end{array}$$

where *I* is the maximum distance between the projected outline points of a particle and *L* is the short axis of the equal-area ellipse when the long axis of the particle is *I*.

(2) $$\begin{array}{c} S=2\sqrt{\frac{\pi A}{P}}\; \end{array}$$

where *A* is the projection area of a particle and *P* is the actual perimeter of the particle’s projected contour.

The gravels were sieved into three particle size groups: 2–5 mm, 5–10 mm, and 10–20 mm. A total of 1000 particles in each particle size group were randomly sieved as experimental samples for the CT and permeability tests. Then, 100 out of the 1000 particles in each particle size group were randomly selected and measured. The sampling number was 10% of the total gravel, which conformed to the statistical sampling principle. Therefore, the mean value of the shape parameter of the 100 sample particles was used as the shape parameter of the same particle group.

We designed a fixture to hold and rotate an individual particle. The measurement process was as follows: First, the fixture was fixed onto a gravel particle’s longest axis and we assumed that the particle was set at an angle of 0°. We then rotated the gravel to 60° and 120°. We used a laser scanner to capture its outer contour at each of three angles and imported these contours to ImageJ. The geometric dimensions of the outer contours required for Equations (1) and (2) were measured using ImageJ and calculated to obtain an individual particle’s aspect ratio and roundness at each of the three angles. The average values of the particle’s aspect ratio and roundness at the three angles were taken as *α* and *S* and had three-dimensional physical significance.

The design process of the test samples’ particle size distributions was as follows: The particle size distribution of 100 particles selected from the 2–5 mm, 5–10 mm, and 10–20 mm size groups was obtained by counting their profiles geometrically and were labeled D_Ⅰ_, D_Ⅱ_, and D_Ⅲ_. Then, D_Ⅰ_, D_Ⅱ_, and D_Ⅲ_ were multiplied by the percentage in Table 2 and combined to obtain three new particle size distributions of 2–20 mm, which were labeled D1, D2, and D3, and were used as the test samples’ particle size distributions.

#### 2.2.2. Proctor Compacion Test

The proctor compaction test examines gravel’s maximum and minimum dry densities. The equipment for this experiment included a compaction cylinder (whose volume was 2103.0 cm^3^), a hammer (whose mass was 2.5 kg), and a guide cylinder. The falling height of the hammer was 457 mm. The maximum porosity of gravel is the porosity when it is in natural accumulation; the minimum porosity of gravel is the porosity when it is in the densest accumulation. The porosity can be calculated from the dry density as follows [45]:

(3) $$\varphi =1-\frac{\rho_{d}}{\rho_{G}}\;$$

where *φ* is the porosity of the gravel, *ρ_d_* is the dry density of the gravel, and *ρ_G_* is the density of the gravel.

#### 2.2.3. Computed Tomography Scanning Test

The CT scanning test was performed on particle packs contained in the permeameter, which was a high-spatial-resolution Siemens 40 CT machine from Changjiang River Scientific Research Institute, to observe the internal pore structure. The image reconstruction matrix was 512 × 512, and the minimum spatial resolution was 0.29 mm.

#### 2.2.4. Constant-Head Permeability Test

**Figure 3:**
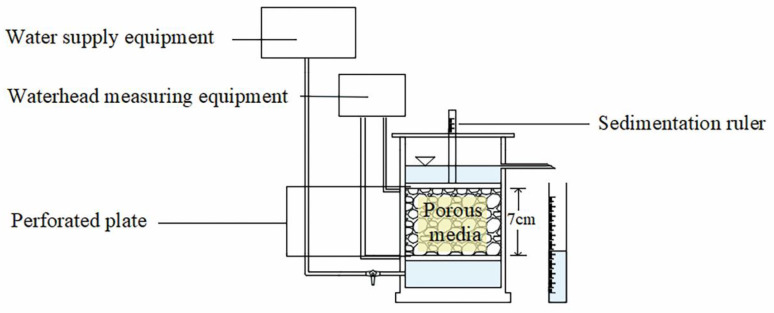
Constant-head permeability test system.

The constant-head permeability test was performed on particle packs after scanning. The test used boiled purified water to eliminate the influence of bubbles. The sample packs were soaked for more than 2 h to saturate entirely before conducting the permeability test. The test flow direction was from the bottom to the top, and the overflow nozzle controlled the downstream waterhead (Figure 3). The waterhead of the water tank was gradually raised to provide a 0.05 hydraulic gradient with a waiting period of 10–20 min for each step. The upstream and downstream waterhead values were recorded, and the flow of the overflow pipe was measured over time with a measuring cylinder. The following hydraulic gradient of the permeability test was conducted as long as the flow measured the same values three consecutive times. In each test, the pack had no seepage deformation, and the sedimentation value measured at the top of the sample was maintained at 0.

### 2.3. The New Method of Surface Area Measurement Based on CT Image Segmentation Using SVM

Before analyzing the pore characteristic of particle packs in CT images, the particles, pores, and containers in the image were segmented separately using SVM. Classifying pixels is the essence of image segmentation. SVM is used for pattern classification and nonlinear regression in multilayer perceptron and principal radial function networks by building a classification hyperplane as the decision surface, which maximizes the isolation edge of positive and negative examples [46]. SVM is the approximate realization of structural risk minimization based on statistics. The learning machine’s generalization error rate on test data is bounded by the sum of the training error rate and an item depending on the Vapnik–Chervonenkis dimension [47]. The SVM’s value for the former item is zero in a separable mode, and the second item is minimized. Therefore, SVM can provide better generalization performance when it comes to pattern classification, which is unique. The discriminant equation of the SVM model is [47]:

(4) $$Y=g\left(X;\omega \right)=\{\begin{array}{c} 1\;\omega^{T}X+b>0\\ -1\;\omega^{T}X+b<0 \end{array}\;$$

where *X* denotes an eigenvector of an arbitrary instance input and *x_i_* is a concrete feature in an eigenvector in which $X=\left(x_{1},x_{2},\ldots ,x_{m}\right)$. The model is trained with all positive instances of labels for which *Y* = −1 to pursue the appropriate values for the parameters *ω* and *b.* Thus, an unknown *X_i_* would be classified as a positive case when $\omega^{T}X_{i}+b>0$, and vice versa.

The C-SVC (a type of SVM solution model) is suitable for binary problem judgment. In the sample, set *T* is the input [48]:

(5) $$T=\left\{\left(x_{1},y_{1}\right).\ldots .\left(x_{l},y_{l}\right)\right\}\in {\left(X\times Y\right)}^{1}\;$$

where *x*_i_ ∈ *X* = R^n^, *y*_i_ ∈ *Y* = {1, –1} (*i* = 1, 2, …, *l)*, and *x_i_* is an eigenvector. After selecting a suitable kernel function K(x,x′) and an appropriate parameter C, we constructed and solved the global optimization:

(6) $$\underset{\alpha }{\min}\frac{1}{2}\overset{j}{\underset{i=1}{\sum }}\overset{l}{\underset{j=1}{\sum }}y_{i}y_{j}\alpha_{i}\alpha_{j}K\left(x_{i},x_{j}\right)-\overset{l}{\underset{j=1}{\sum }}\alpha_{j}\;$$

(7) $$s.t.\;\overset{l}{\underset{i=1}{\sum }}y_{i}\alpha_{i}=0,0\leq \alpha_{i}\leq C,i=1,\ldots ,l\;$$

Therefore, the optimal solution α* is α* = (*α*_1_*,…, α_l_*)^T^. We selected a positive element of vector α* (0 < *α*_j_ < *C*) and calculated the threshold b*:

(8) $$b^{*}=y_{i}-\overset{l}{\underset{i=1}{\sum }}y_{i}\alpha_{i}^{*}K\left(x_{i}-x_{j}\right)\;$$

Then, we constructed the decision function *f(x)*:

(9) $$f\left(x\right)=sgn(\overset{l}{\underset{i=1}{\sum }}\alpha_{i}^{*}y_{i}K\left(x,x_{i}\right)+b^{*})\;$$

**Figure 4:**
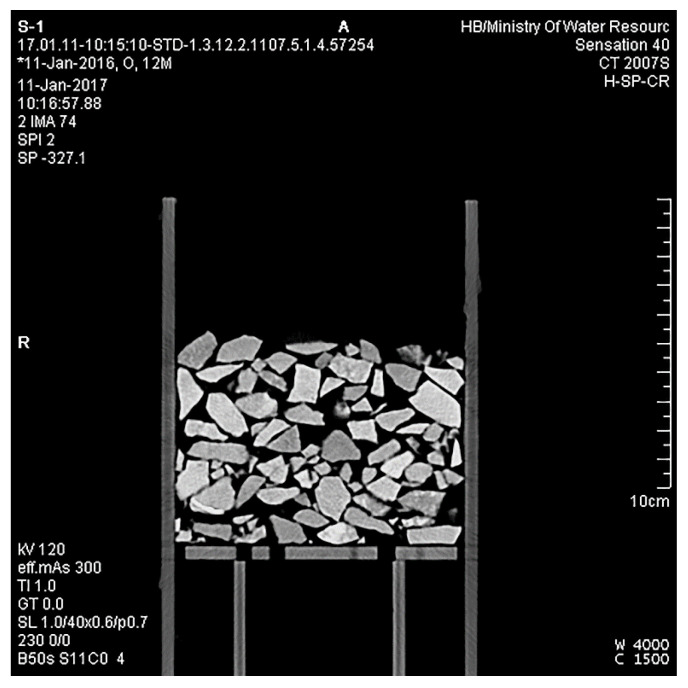
A CT image of S1.

We classified the pixel values of different objects in CT images first. A CT image of S1 is given as an example in Figure 4. The pores between particles in the CT image are black, while the particles and the container are white with inhomogeneous saturation levels. The pixel values of particles and pores were set as training samples in the limited area according to the SVM method (Equations (4)–(9)) using a code we compiled in MATLAB. The 8-bit image data picture is a three-dimensional matrix with 484 rows, 484 columns, and 3 pages.

The material’s interactive medical image control system (MIMICS) was used to reconstruct and measure the 3D model’s volume and surface area after segmenting the CT images. The pores and particles were reconstructed separately. The reconstruction method was gray value interpolation [49], which considers the partial volume effect. The advantage of gray value interpolation is that it gives lots of detail and the correct dimensions. The validity of the surface area measurement of the reconstructed pore network model was verified by comparing the porosity of the reconstructed particle pack model with the same test sample using the relative error. The porosity of the reconstructed particle pack model is as follows:

(10) $$\varphi_{sim}=\frac{V_{pore}}{V_{0}}\times 100\%\;$$

where *φ_s_*_im_ is the porosity of the pore network model, *V_pore_* is the volume of the pore network model, and *V_0_* is the sum of the volume of the pore network model and particle pack model. The relative error of the comparison of the porosity of the reconstructed particle pack model with the same test sample is as follows:

(11) $$R=\frac{\left|\varphi_{sim}-\varphi_{Lab}\right|}{\varphi_{Lab}}\times 100\%\;$$

where *R* is the relative error and *φ*_Lab_ is the porosity of the test sample. A smaller *R* means that the two quantities being compared are closer.

### 2.4. Fractal Analysis Method

The pore–particle interface of porous media has good fractal characteristics, and the fractal dimension can be used to study the pore structure’s complexity quantitatively using the box-counting method [50]. Assume that the objects are covered by orthogonal line grids with an increasing lattice constant. The number (*N*) of those grids (boxes) that contain any part of the structure is calculated by the size of each box (equal to the lattice constant *ε*) and stored in the data list. The macro increases the box size (*ε*) in selectable step sizes; for each box size, any boxes that contain at least 1 pixel of the contour line were counted (*N*). This count depends on the box size *ε*, box-counting b, length *L*, and fractal dimension *D* according to Equation (12) [50]:

(12) $$N\varepsilon \propto \varepsilon^{-D}\;$$

Thus, for fractal objects, a double-logarithmic plot yields a straight line:

(13) logN(ε)=−Dlogε+c 

where *D* can be determined as the absolute value of its slope, and the constant c describes the ordinate intercept. Binary image processing is required before calculating the box dimension of the pore–particle interface (*D*). The binary image is divided by an equivalent grid with a side length *ε*, where the grid occupied by white pixels is defined as N(*ε*). Thus, lg*N*(*ε_i_*)/lg(1/*ε*)→*D* when *ε*→0. For a specific decreasing sequence {*ε_i_*}, the definition of the fractal dimension approximates its slope. The decreasing sequence {*ε_i_*} is usually defined via a dichotomy. Then, the box-counting dimension can be defined as follows:

(14) $$D=\underset{\varepsilon \to 0}{lim}\frac{lgN\left(\varepsilon \right)}{lg(1/\varepsilon )}\;$$

For boxes with different side lengths (*ε*), the required number of boxes (N(*ε*)) to cover the pore pixels varies. A gray pixel can be covered only by a box with a specific side length considering the relativity of a binary gray image. For the linear equation, the least squares method was used to fit the data points linearly:

(15) lgN(ε)=a⋅[−lg(1/ε)]+b 

where the slope *a* is dimension *D*.

We compiled a code in MATLAB to calculate the box dimension of the pore–particle interface in CT images of test samples according to Equations (12)–(15). Each sample had 140 CT images.

### 2.5. Pore Scale Simulation

**Figure 5:**
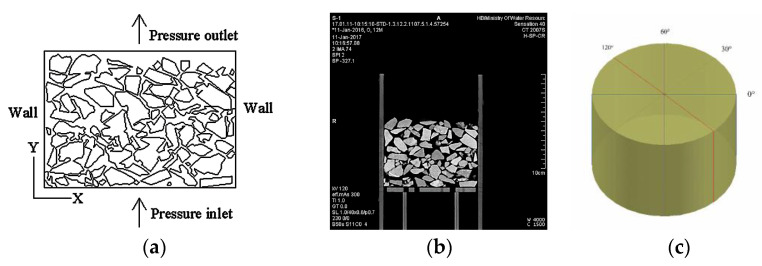
Numerical simulation model. (**a**) Schematic of the computational domain and the boundary. (**b**) The corresponding CT section of S1. (**c**) Schematic representation of the model’s cut location.

The water flow in particle packs was simulated in FLUENT. Figure 5 shows a schematic of the computational domain and boundary conditions in the numerical simulations consisting of the CT image. We took four central cross-sectional CT images of each pack for the simulation (Figure 5c). The calculation area was 10 × 7 cm. The CT sections were exported to the DXF format file using MIMICS to build the model and generate regions using AutoCAD. The pressure inlet was determined using the corresponding value obtained from the permeability test while the outlet was free flow. Both the sidewalls and particles were impermeable.

The control equation of the simulation in the domain area *Ω* is given below [51].

For velocity ***v***: *Ω* → R^2^; and pressure *p*: *Ω* → R:

(16) $$\{\begin{array}{c} \begin{array}{c} \begin{array}{c} \rho \dot{v}+\rho \left(v\cdot \nabla \right)v-\mu \nabla^{2}v+\nabla p=g\;\mathrm{in}\;\Omega \\ \nabla \cdot v=0\;\mathrm{in}\;\Omega \\ \;v=\bar{v}\;\mathrm{on}\;\Gamma_{D}\\ \;\sigma \cdot n=\bar{t}\;\mathrm{on}\;\Gamma_{N} \end{array}\\ v\left(\cdot ,0\right)=v_{0}\;\mathrm{in}\;\Omega \end{array}\\ p\left(\cdot ,0\right)=p_{0}\;\mathrm{in}\;\Omega \end{array}$$

where $\dot{v}=\partial v/\partial t$, ∇ is the gradient operator, *ρ* is the density of the fluid, *μ* is the dynamic viscosity of the fluid, **g** is the body force, **n** is the unit outward normal on the boundary *Γ* of *Ω*, *Γ*_D_ is the part of the boundary that experiences the Dirichlet boundary condition, $\bar{v}$ is the applied velocity, *Γ*_N_ is the part of the boundary that experiences the Neumann boundary condition, and $\bar{t}$ is the applied time. Here, *Γ = Γ*_D_ ∩ *Γ*_N_ and *Γ*_D_ ∩ *Γ*_N_ = ∅. ***v***_0_ and *p*_0_ are the initial velocities and pressure fields in the fluid in the domain, respectively. The pseudo-stress ***σ*** is given as follows:

(17) σ=μ∇v−pI

where ***I*** is the second-order identity tensor.

The code uses an unstructured quadrilateral grid of the finite volume method, which has excellent adaptability. The calculation of the time-dependent physical quantities was transient. The number of grids in the calculation area was more than 50,000; therefore, the pressure field was calculated based on staggered grids using the semi-implicit method for pressure-linked equations (SIMPLE) to solve Equations (16)–(17) in order to reduce the calculation time. The models were saturated, and only the solid and liquid phases were in the calculation area.

The inlet water pressure in the model was consistent with that of the laboratory test. The flow state in the simulation was laminar flow; therefore, the velocity ratio at the outlet of the same model to the hydraulic gradient was invariant under different hydraulic gradients. This was defined as the hydraulic conductivity of the model.

The validity of the simulation was verified by comparing the hydraulic conductivity of the simulation and the results of the constant-head permeability test. The root-mean-square error (RMSE) [52], Pearson correlation coefficient (PCC) [53], and Nash–Sutcliffe model efficiency coefficient (NSE) [54] were used to evaluate the accuracy of the simulation results:

(18) $$RMSE=\sqrt{\frac{1}{m}\overset{m}{\underset{i=1}{\sum }}{\left(k_{Lab,\;i}-k_{Sim,i}\right)}^{2}}\;$$

(19) $$PCC=\frac{\sum_{i=1}^{m}\left(k_{Lab,i}-{\bar{k}}_{Lab}\right)\left(k_{Sim,i}-{\bar{k}}_{Sim}\right)}{\sqrt{\sum_{i=1}^{m}{\left(k_{Lab,i}-{\bar{k}}_{Lab}\right)}^{2}}\sqrt{\sum_{i=1}^{m}{\left(k_{Sim,i}-{\bar{k}}_{Sim}\right)}^{2}}}\;$$

(20) $$NSE=1-\frac{\sum_{i=1}^{m}{\left(k_{Lab,i}-k_{Sim,i}\right)}^{2}}{\sum_{i=1}^{m}{\left(k_{Lab,i}-{\bar{k}}_{Lab}\right)}^{2}}\;$$

where *m* is the number of data points; *k_Lab_*_,*i*_ and *k_Sim_*_,*i*_ are the ith experimental and simulated *k*, respectively; and ${\bar{k}}_{Lab}$ and ${\bar{k}}_{Sim}$ are the equivalent experimental and simulated mean *k*, respectively. The RMSE can vary from 0 to +∞. A smaller RMSE indicates a better-simulated data fit for the experimental data. The PCC varies from −1 to 1, where higher values indicate better data congruence. The NSE varies from −∞ to 1 and can assess hydrological models’ predictive power, where a value closer to 1.0 indicates a better match between the observed and modeled values.

We used Tecplot to process the calculation results from FLUENT in order to generate flow field diagrams. The velocity field is a vector field that describes a fluid’s velocity distribution at several points in space. The velocity field is constant when the fluid flows stably and does not change over time.

## 3. Results and Discussion

### 3.1. Gravel Pack Shape, Particle Size Distribution, and Porosity

**Figure 6:**
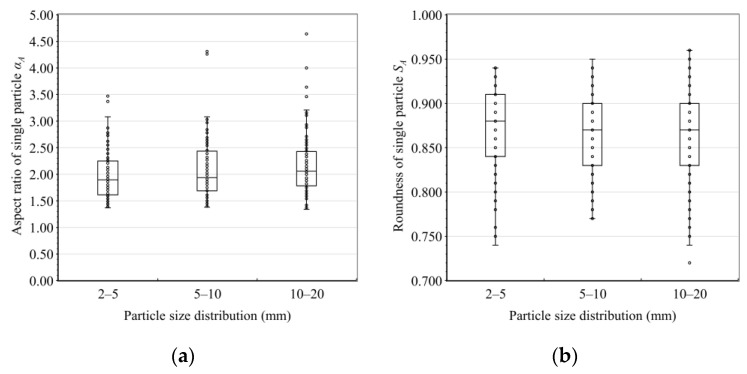
Box plots of the shape parameters. (**a**) The box plot of the aspect ratios. (**b**) The box plot of the roundnesses.

**Figure 7:**
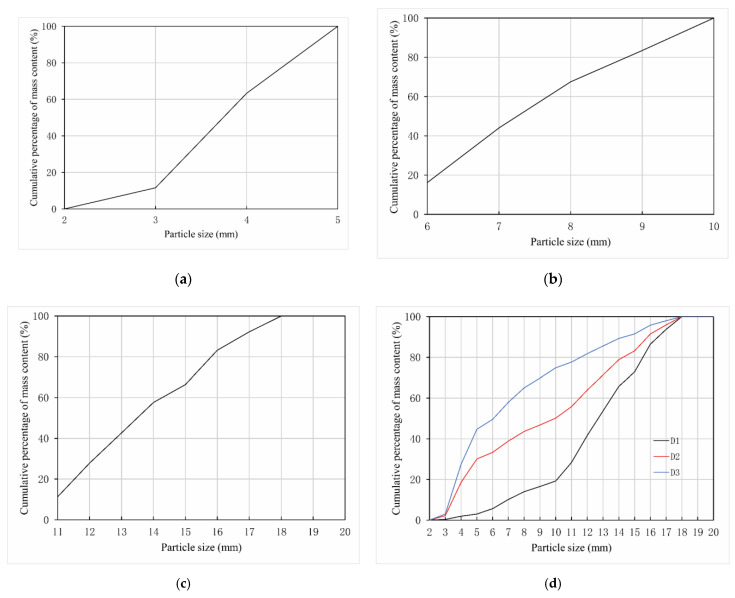
Particle size distributions of the gravel. (**a**) D_Ⅰ_, size range was 2–5 mm. (**b**) D_Ⅱ_, size range was 5–10 mm. (**c**) D_Ⅲ_, size range was 10–20 mm. (**d**) D1, D2, and D3 particle size distributions in the laboratory test.

Box-plots of the *α* and *S* of the 300 sampled particles from the different size groups are shown in Figure 6. The figure indicates that the distribution of the particles’ *α* was positively skewed, while that of *S* was negatively skewed. The mean value of *α* and *S* of 100 samples in each size group was taken as the gravel’s shape parameter in this size group, which were recorded as *α_A_* and *S_A_*, respectively. The D_Ⅰ_, D_Ⅱ_, and D_Ⅲ_ particle size distributions of the sampled gravels are shown in Figure 7a–c. The particle size distributions D1, D2, and D3 of the particle packs after the CT scanning and permeability tests, following the method described in Section 2.2.1, are shown in Figure 7d.

**Figure 8:**
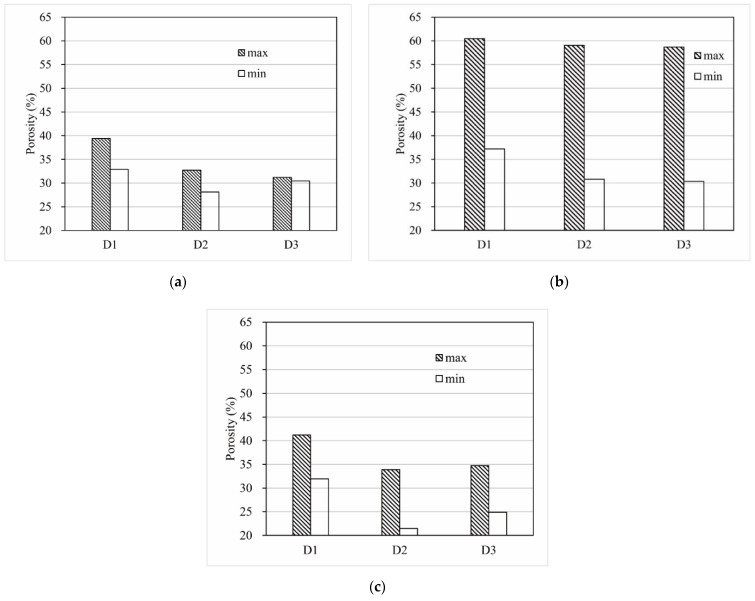
The porosities of the packs. (**a**) The porosities of the glass ball packs. (**b**) The porosities of the gravel packs. (**c**) The porosities of the plastic octahedron packs.

Figure 8 depicts the maximum and minimum porosities of the samples with D1, D2, and D3 particle size distributions according to the results of the proctor compaction test. As can be seen from the bar graphs, the porosity range variation was the most significant for gravels and the most minor for glass balls for the same particle size distribution. Therefore, the porosity of packs should be determined using the glass balls’ porosity range. The porosities of the samples in D1, D2, and D3 were set to 38.81% (P1), 32.29% (P2), and 31.22% (P3), respectively.

Because the particle size distribution of the test samples was obtained by multiplying the particle content of the sampled particle size distributions of 2–5 mm, 5–10 mm, and 10–20 mm by the percentage in Table 2, the aspect ratio of a particle pack (*α_P_*) was equal to the sum of *α_A_* for each particle size distribution multiplied by the percentage in Table 2. Similarly, the roundness of a particle pack (*S_p_*) was equal to the sum of *S_A_* for each particle size distribution multiplied by the percentage from Table 2. The particle shape parameters of the glass ball pack and plastic octahedron pack were fixed values that were independent of the size distribution.

Information on the test samples is listed in Table 3.

### 3.2. Effect of the Particle Pack Shape on the Pore Characteristics of Gravels

#### 3.2.1. CT Image Segmentation

**Figure 9:**
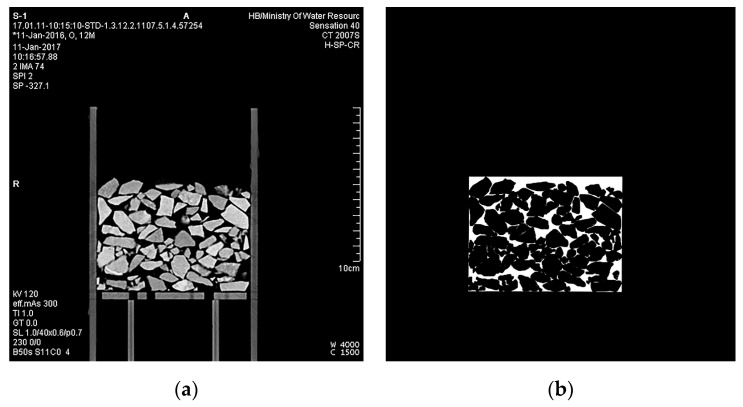
Image segmentation based on the SVM. (**a**) The original CT scanning image. (**b**) The image after segmentation.

Figure 9 shows the segmentation result of a CT image from S1 using SVM. As shown in Figure 7b, the outlines of pores and particles after image segmentation were clear, demonstrating that the image segmentation code was effective. All CT images were processed in batches. Due to the significant number of CT images of all samples, only the segmentation results of one image are shown here. The edges of pores in the other segmented images are also precise.

**Figure 10:**
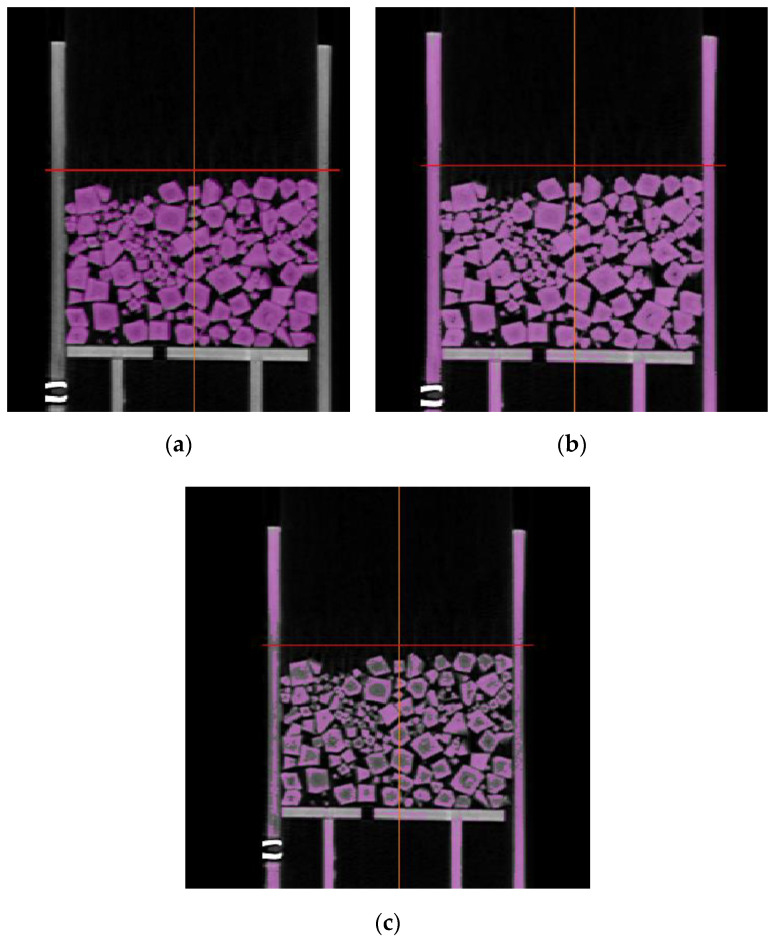
Results of the segmentation of the same CT image of O1 via different methods. (**a**) The SVM method. (**b**) The gray-scale morphology method. (**c**) The histogram segmentation method.

There is currently no standard for verifying the validity of image segmentation results. The outline of the segmented target can only be observed by the human eye to see whether it is clear and complete. The accuracy of calculating the pore–particle interface’s fractal dimension and the pore surface area depends entirely on the accuracy of the image segmentation. Therefore, we verified the validity of the SVM by comparing the segmentation results of the same CT image using SVM, the gray-scale morphology method [55], and the histogram method [56]. Gray-scale morphology and histogram segmentation are common segmentation methods. Figure 10 shows the comparison of O1′s CT image segmentation result. Among the three materials, the CT image segmentation of plastic octahedrons was the most difficult because of its non-uniform color in the CT images due to the uneven particle density. The plastic particles had a high density at the edges and a low density in the middle, which could not be avoided when manufacturing due to their pouring process. In addition to the problem of the non-uniform density of plastic particles, the densities of the plastic particles and the resin permeameter were close, which was also an obstacle. In Figure 10, purple represents the particles segmented from the image. The original CT image was used as the background to discriminate the segmentation effect. The gray-scale morphology method could not separate the particles from the permeameter in the CT image of O1, according to Figure 10b. Using the histogram method not only failed to separate the particles from the permeameter but also over-divided the particles in the same image, according to Figure 10c. This showed that the SVM method segmented the CT image of O1 with significantly better results than the other two methods. SVM had advantages when segmenting the same heterogeneous object or different objects with similar densities.

#### 3.2.2. Fractal Analysis of the Pore–Particle Interface Based on CT Images

**Figure 11:**
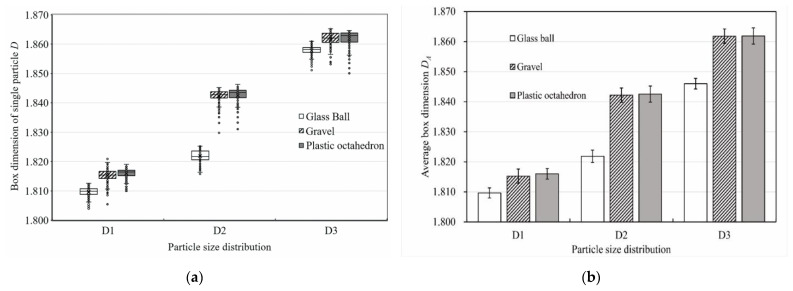
Box dimensions of the pore–particle interfaces. (**a**) The box plot of the box dimensions of single particles’ pore–particle interfaces. (**b**) The bar graph of the packs’ average box dimensions. The figure’s error lines were based on the standard deviation.

Figure 11 illustrates the change in the box dimension of the pore–particle interface with different particle pack shapes. According to Figure 11a, the box dimension of the pore–particle interface (*D*) in each CT image of all the packs ranged from 1.804 to 1.865. All box dimensions of the pore–particle interface were above their topological dimension (the value of which is 1) and less than two dimensions (the value of which is 2), demonstrating that the pore–particle interface of the samples showed prominent fractal characteristics based on the theory of Mandelbrot [30]. The median line of boxes was not located in the middle of the box, indicating that the distribution of *D* of each sample presented a skewed distribution. The points outside the box in the figure are outliers. The reason was that the CT machine scanned the sample spiral forward for slice scanning, and some particles in the CT image appeared to be suspended without contact. Particles came into contact in three–dimensional space; however, the two-dimensional slice may not cut to the contact point, which is a normal phenomenon. When calculating the average box dimension of each sample’s pore–particle interface (*D_A_*), these outliers must be deleted before the calculation. The calculation results are shown in Figure 11b. Unless otherwise specified, later box dimensions mentioned refer to the average box dimensions. Except for the standard deviation of the *D_A_* of the octahedron pack for the D2 and D3 particle size distributions being 0.003, the standard deviation of the *D_A_* of the other packs was 0.002. As can be seen from the second bar chart, the *D_A_* of the ball pack was the smallest, and there was little difference between the *D_A_* of the gravel pack and that of the octahedron pack for the same particle size distribution. For the D1 particle size distribution, the *D_A_* of the ball pack was 1.810. For the D2 particle size distribution and D3 particle size distribution, the *D_A_* values of the glass ball pack were 1.822 and 1.846, respectively. The *D_A_* values of the gravel pack and octahedron pack were 0.31% and 0.64% larger than that of the ball pack for the D1 particle size distribution, respectively; the *D_A_* values of the gravel pack and octahedron pack were 1.12% and 1.14% larger than that of the ball pack for the D2 particle size distribution, respectively; both the *D_A_* values of the gravel pack and the octahedron pack were 0.86% larger than that of the ball pack for the D3 particle size distribution. For the D2 particle size distribution, the content of fine particles (less than 5 mm) in the packs ranked second among the three particle size distributions. However, for the D2 particle size distribution, the difference between the *D_A_* of the ball pack and the *D_A_* of the other two shaped particle packs was the largest.

**Figure 12:**
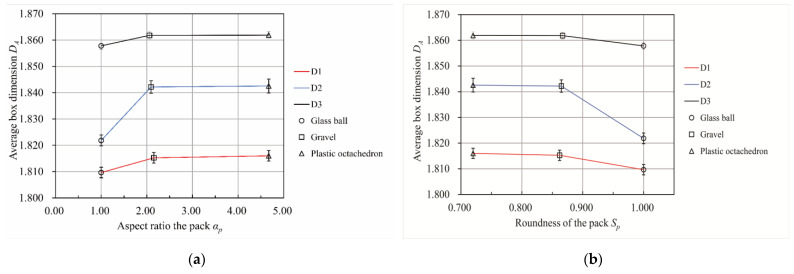
The relationship between the particle pack shape and the average box dimension of the pore–particle interfaces. The error lines in the figures were based on the standard deviation. (**a**) The relationship between the average box dimension of the pore–particle interface and the aspect ratio of the packs. (**b**) The relationship between the average box dimension of the pore–particle interface and the roundness of the packs.

Figure 12 describes the change in the average box dimension of the pore–particle interface with a change in the shape of the particle pack for three particle size distributions. According to Figure 10a, *D_A_* increased with an increase in *α_p_* when *α_p_* was less than 2.154. *D_A_* remained almost stable when *α_p_* was larger than 2 and less than 5. As shown in Figure 10b, *D_A_* decreased with a decrease in *S_p_* when *S_p_* was larger than 0.862 and less than 1. *D_A_* remained nearly unchanged when *S_p_* was larger than 0.72 and less than 0.862. Both figures reflect that the influence of the particle pack shape parameters on *D_A_* was the most significant for the D2 particle distribution.

**Figure 13:**
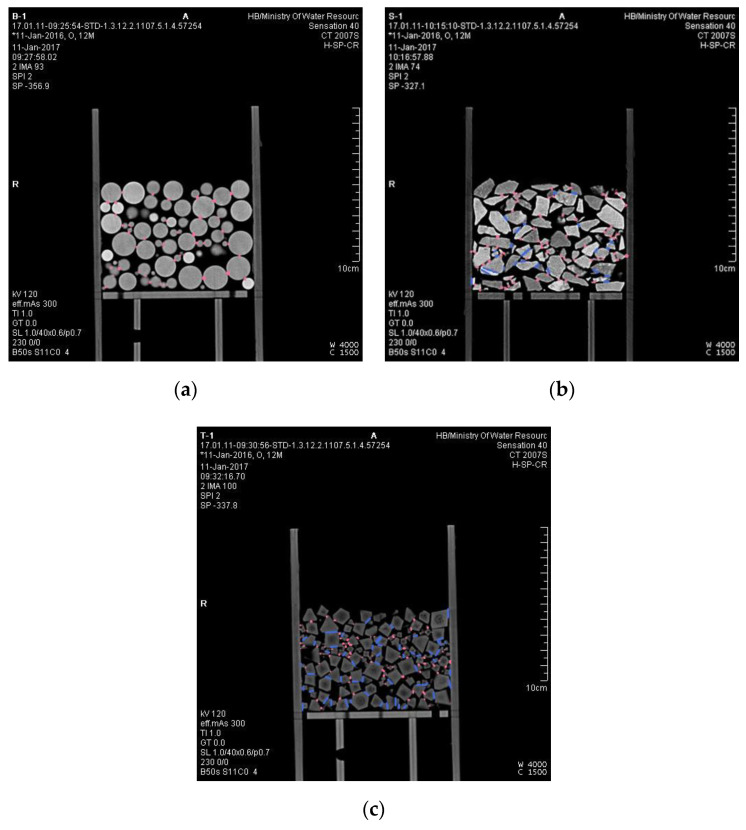
Contact markers on CT images. (**a**) CT middle section of B1. (**b**) CT middle section of S1. (**c**) CT middle section of O1.

Figure 13 shows the CT image of the middle vertical sections of B1, S1, and T1. In the figure, pink marks the point—to—point contact relationships and blue marks the edge-to-edge contact relationships. The length marked in the figure is not the actual contact length. The format of Figure 13 is TIFF, which is not the standard format of CT images used for display in other papers. In fact, CT images come in a DICOM format, which has a higher resolution and can only be read using special CT image editing software. We annotated and counted the contact relationship of CT images in MIMICS. We defined a particle contact length of less than 1 mm as a point—to—point contact relationship and a length greater than 1 mm as an edge-to-edge contact relationship. In Figure 13, B1 has 42 point—to—point contact relationships, S1 has 68 point—to—point contact relationships and 24 edge-to-edge contact relationships, and O1 has 93 point-to-point contact relationships and 54 edge—to—edge contact relationships. CT images can only show the two-dimensional contact of particles. In fact, in three—dimensional space, there were also edge—to—surface contact relationships in the gravel pack and octahedral pack. We calculated the type and quantity of contact relationship of all CT images of nine test samples. There was only point-to-point contact relationship between spheres, which made it the most special. Under the same condition, compared with other shaped particle packs, the number of contact points between spheres was the least. From the perspective of the pore space, the pore structure of spheres was the simplest, and the average box dimension of the pore–particle interface was the smallest. Once the particles deviated from the sphere, the number of contact points soared due to there being more contact relations between particles. This led to the average box dimension of the pore–particle interface and pore structure complexity skyrocketing. When the shape parameters of particle packs reach a critical value, the box dimension of the pore–particle interface reaches a plateau. Unfortunately, we did not find that critical value due to the few kinds of materials used in the laboratory test. We will research the critical value through numerical simulation in future studies.

Li et al. [57] established a new method for evaluating the complexity of digital rock pore structure using the relative value of the box dimension and verified this method’s effectiveness by calculating some stones’ CT images. They set up a series of rock models with porosities of 0.05 to 0.4 and calculated the three-dimensional box dimension of the corresponding pore–rock interface in the range of 2.2–2.6. Their calculation results showed that when the rock’s porosity was 0.05–0.2, the box dimension increased sharply with a rise in porosity. However, when the porosity exceeded 0.2, the box dimension grew slowly. This indicated that there was a critical porosity. When the porosity of particles exceeded the critical porosity, the box dimension increased slightly. The box dimension under the critical porosity can be used as a parameter to judge the complexity of the pore structure. Although our model and method differed from Li’s calculation, we obtained the same law regarding the box dimension. As shown in Figure 8, when the three shapes of particle packs with the same particle size distribution had their minimum porosity, the porosity ranking of the samples was as follows: porosity of the sphere pack > porosity of the gravel pack > porosity of the plastic octahedron pack. This indicated that the larger the particle pack’s aspect ratio, the smaller the porosity. The relationship between the particle pack’s roundness and particles’ porosity was the opposite. The average box dimension we calculated was the average of the box dimensions of all CT images of each sample, which is a parameter that was between two-dimensional and three-dimensional. The relationship between the average box dimension of the pore–particle interface and the particle pack shape parameters in Figure 12 showed that there was a critical shape parameter, and the average box dimension corresponding to the critical shape parameter could be used as the basis for judging the complexity of the pore structure and evaluating the particle pack shape of the gravel. Our calculations were based on the CT images of actual particle packs, while Li used numerical models. Our conclusions were consistent because of the relationship between the particle shape parameters and the porosity. Wang et al. [58], Han et al. [59], Xiu et al. [60], and Ari et al. [61] also reported similar conclusions when using simulations. We illustrated the existence of the critical box dimension from the actual particle pack and verified their conclusions via a laboratory experiment.

#### 3.2.3. Analysis of the Specific Surface Area of the Reconstructed Pore Network Model

**Figure 14:**
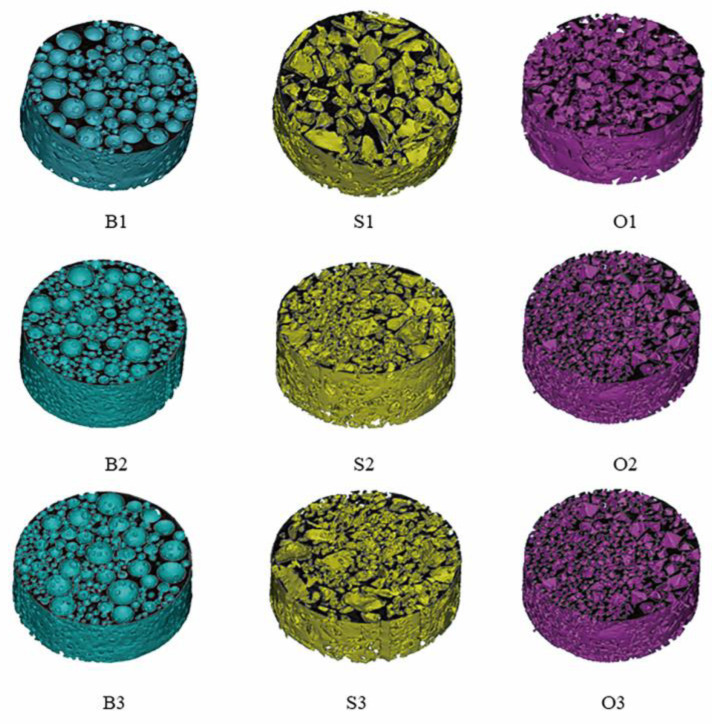
The pore network model of the packs. All models were cut in the middle to show their internal structure.

After the image segmentation, the pore images were imported into MIMICS to generate a 3D model (as shown in Figure 14) of the pore network of packs. We used porosity as the determination criterion to verify the accuracy of the pore model according to Equations (10) and (11), as shown in Table 4. In Table 4, all the relative errors between the porosity of the model and the sample were less than 4%, which implied that the relative errors between the model’s surface area and the sample’s surface area were also less than 4%. This demonstrated that the method used to measure the surface area of porous media in Section 2.3 was appropriate.

**Figure 15:**
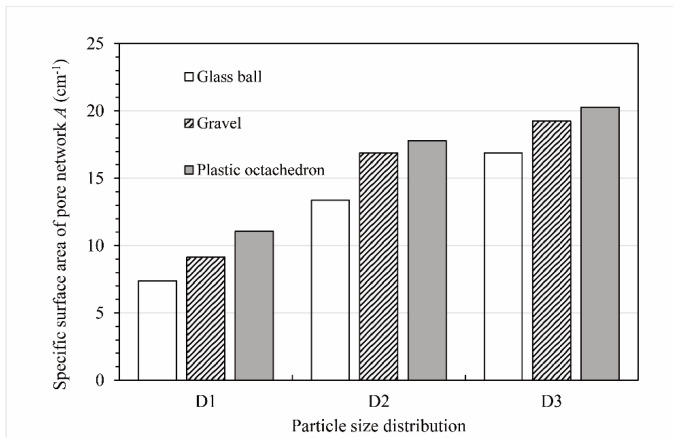
Specific surface area of the pore network (*A*) values of the different packs.

Figure 15 shows the specific surface area of the pore network (*A_p_*) values of all the packs. As can be seen from this graph, the *A_p_* of the ball pack was the smallest for each particle size distribution. For the D1 particle size distribution, the *A_p_* of the ball pack was 7.39 cm^−1^. For the D2 particle size distribution and D3 particle size distribution, the *A_p_* values of the ball packs were 13.37 cm^−1^ and 16.88 cm^−1^, respectively. Moreover, the *A_p_* values of the gravel pack and octahedron pack were 23.7% and 49.7% larger than that of the ball pack for the D1 particle size distribution, respectively; the *A_p_* values of the gravel pack and octahedron pack were 26.4% and 33.1% larger than that of ball pack for the D2 particle size distribution, respectively; and the *A_p_* values of the gravel pack and octahedron pack were 14.0% and 20.1% larger than that of the ball pack for the D3 particle size distribution, respectively. The *A_p_* of the octahedron pack was the largest for each particle size distribution. In particular, the difference between the *A_p_* of the octahedron pack and that of the ball pack was the largest for the D1 particle size distribution. The *A_p_* of the gravel pack was the smallest for the D1 particle size distribution and the largest for the D3 particle size distribution. The *A_p_* values of the ball pack and octahedron pack also showed the same trend.

**Figure 16:**
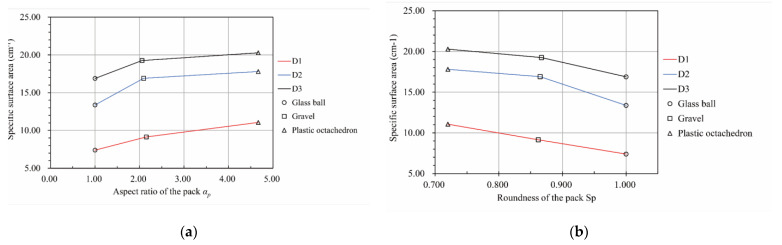
The relationship between the particle pack shape and the specific surface area of the pore network of the pack. (**a**) The relationship between the specific surface area of the pore network and the aspect ratio of the pack. (**b**) The relationship between the specific surface area of the pore network and the roundness of the pack.

Figure 16 demonstrates that the pore network’s specific surface area varied with the particle pack’s shape for three particle size distributions. According to Figure 16a, the specific surface area of the pore network increased with an increase in the aspect ratio of the particle pack. Conversely, there was a downward trend in the specific surface area of the pore network with the growth in the roundness of the particle pack based on Figure 16b.

The KC formula and other modified forms [6,13,14,15,16,62,63,64] require the surface area of particles rather than the surface area of the pore network to predict the permeability coefficient. However, the fluid flows along the particle surfaces and the calculation area’s side wall. The KC formula and other modified forms ignore the flow along the side wall, resulting in a smaller calculated hydraulic conductivity. Considering the influence of the sample volume on the pore surface area, we suggest using the surface area of the pore network per unit volume, i.e., the specific surface area of the pore network, to predict the permeability of the gravel. Furthermore, those formulas are built through simulation, which is difficult to verify and use directly in laboratory and field tests. The new method we proposed to measure the surface area of the pore network of real gravel was reliable and is expected to increase the practicality of these formulas.

### 3.3. Effect of the Particle Pack Shape on the Permeability of Gravel

#### 3.3.1. Constant-Head Permeability Test Result

**Figure 17:**
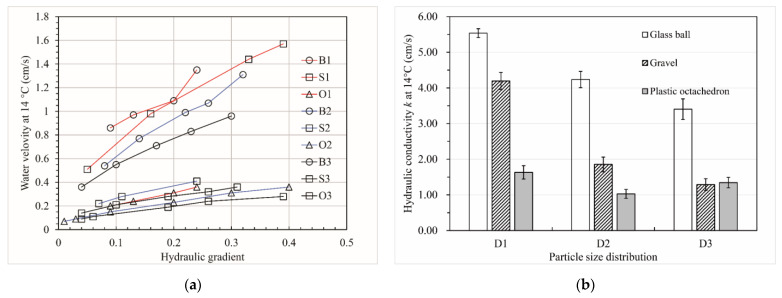
Permeability test results of the packs. (**a**) The relationship between the hydraulic gradient and the flow velocity of the pack. (**b**) The hydraulic conductivity of the pack. The error bars in the figure were based on the standard deviations.

Figure 17 illustrates the results of the constant-head permeability tests of the packs. The first line graph shows the relationship between the water velocity (*V*) and the hydraulic gradient (*J*) at the test water temperature (which was 14 °C). In the test, the flow velocity of all packs was stable, and the relationship between the flow velocity and the hydraulic gradient was basically linear. The water flow in the particles was laminar, and the hydraulic conductivity (*k*) could be calculated using the Darcy formula. The particles did not move, and there was no seepage deformation in the test. In Figure 15a, when the hydraulic gradient was less than 0.1, the calculated hydraulic conductivity differed from that calculated for other gradients. When debugging the instrument, we found that this was due to the limited accuracy of the water head measuring instrument when the hydraulic gradient was less than 0.1. Therefore, we eliminated the velocity corresponding to a hydraulic gradient of less than 0.1. The number of velocity groups of the samples was sufficient after applying the criterion that the hydraulic gradient needed to be larger than 0.1. The hydraulic conductivity of the samples is shown in Figure 15b. As can be seen from the bar graph, for the D1 particle size distribution, the hydraulic conductivity of the ball pack was 0.32 times and 2.39 times higher than those of the gravel pack and octahedron pack, respectively. Additionally, for the D2 particle size distribution, the hydraulic conductivity of the ball pack was 1.28 times and 3.12 times higher than those of the gravel pack and octahedron pack, respectively. For the D3 particle size distribution, although the hydraulic conductivity of the ball pack was more than 1.5 times higher than those of the gravel pack and octahedron pack, there was a slight difference between the hydraulic conductivity of the gravel pack and the octahedron pack. Therefore, we believe that when the content of fine particles (particle size less than 5 mm) was in a particular range with the same particle size distribution and porosity, the main factor that affected the hydraulic conductivity of porous media is the particle shape. However, with an increase in the fine particle content, the effect of the particle shape on the permeability of porous media decreased (except for spheres).

**Figure 18:**
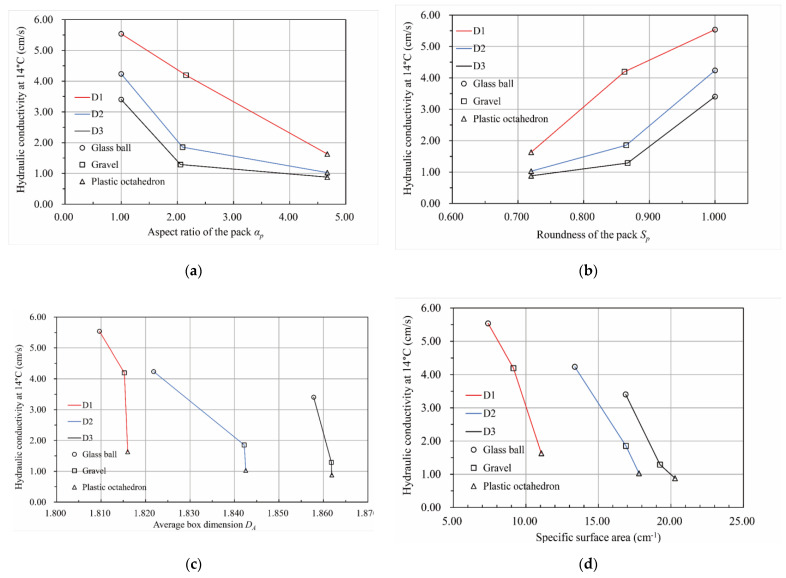
The relationship between the hydraulic conductivity and the pore characteristics of packs. (**a**) The relationship between the aspect ratio of the pack and the hydraulic conductivity. (**b**) The relationship between the roundness of the pack and the hydraulic conductivity. (**c**) The relationship between the average box dimension of the pore–particle interface and the hydraulic conductivity. (**d**) The relationship between the specific surface area of the pore network and the hydraulic conductivity.

Figure 18 illustrates the relationship between the hydraulic conductivity and the pore characteristics of the packs. According to Figure 18a, there was a downward trend in the hydraulic conductivity with an increase in the aspect ratio of the particle pack for the same particle size distribution. The hydraulic conductivity of the same material pack was the largest for the D1 particle size distribution and the smallest for the D3 particle size distribution, which is consistent with the Hazen formula [4], Kozeny formula [5], Kozeny–Carman formula [6], and Terzaghi formula [7]. For the D1 particle size distribution, the hydraulic conductivity of the packs seemed to decrease uniformly with an increase in the particle pack’s aspect ratio. However, for the particle size distribution of D2 and D3, the hydraulic conductivity of the packs decreased gradually when the aspect ratio of the particle pack was larger than 2.1. According to Figure 18b, the relationship between the packs’ hydraulic conductivity and roundness was the opposite. Moreover, the hydraulic conductivity of the packs grew significantly when the roundness of the pack was larger than 0.86 for the particle size distribution of D2 and D3. Meanwhile, for the D1 particle size distribution, the hydraulic conductivity of the packs rose uniformly with an increase in the roundness of the pack. Therefore, similar to the conclusion drawn from Figure 18a, when studying the influence of the particle shape parameters on the permeability of the gravel, packs with different fine particle contents differed from one another. Figure 18c depicts the variation in the hydraulic conductivity of the packs with the average box dimension of the pore–particle interface. As can be seen from this line graph, the hydraulic conductivity of the packs decreased with an increase in the average box dimension of the pore–particle interface for the same particle size distribution. For the D2 particle size distribution, the variation in hydraulic conductivity with the average box dimension of the pore–particle interface from 1.822 to 1.842 was less than those of others. We think this was because the spatial distribution of particles was random and uneven. Coarser pore channels were formed in the middle of some particles in the S2 sample, resulting in its hydraulic conductivity deviating from those of the other packs. As shown in Figure 18d, the hydraulic conductivity of the packs for the same particle size distribution decreased with an increase in the specific surface area of the pore network. The three lines in this figure are approximately parallel, which makes them significantly different from the second line in Figure 18c. Therefore, when studying the relationship between pore characteristics and permeability of the porous media, multiple parameters describing pore characteristics should be used to describe its characteristics comprehensively.

Our results are in agreement with those of Zakhari et al. [65], Liu et al. [66], Conzelmann et al. [67], and Li et al. [68]. In addition to the particle pack shape, we also found that the content of fine particles less than 5 mm in the porous media dramatically influenced a shape’s sensitivity to permeability. We believe this was because there were different contact relationships between particles with different shapes. Contact relationships can be divided into five types: point-to-point, point-to-face, edge-to-edge, edge-to-face, and face-to-face. The sphere pack only had the point-to-point contact relationship, according to Figure 13. With an increase in the aspect ratio or a decrease in the roundness of the particle packs, the other four contact relationships appeared. Generally, the two relationships of edge-to-edge and edge-to-face appeared more in the frontal body pack and less in naturally formed particle packs, such as the gravel pack. Different contact relationships and the number of each relationship affected the pore structure characteristics of the porous media, resulting in a difference in permeability. Some scholars think that the influence of the shape on the permeability of porous media is negligible [18]. We believe that it depends on the different types and number of contact relationships and the fine particle content. For porous media with less fine particle content, such as the D1 grading in our test, it was evident that the influence of shape on permeability could not be ignored. Our laboratory test results implied that the type and number of contact relations affected the shape sensitivity to permeability. The influence of shape on permeability can be ignored in a particular range, but the shape needs to be considered beyond this range. When the shape factor is considered in the formula for predicting permeability, the conditions for the type and number of contact relations should be given. That is also why some scholars add the shape factor to the formula to predict hydraulic conductivity, but the results are only consistent with their experiments or simulations.

#### 3.3.2. Simulation

As was mentioned in Section 3.2, the instrument for measuring the water head was inaccurate when the hydraulic gradient was less than 0.1. Therefore, when using FLUENT to simulate the laboratory test, we only simulated a flow field with a hydraulic gradient ranging from 0.1 to 0.3.

The RMSE, PCC, and NSE values, according to Equations (18)–(20), are listed in Table 5. As can be seen from the table, the maximum RMSE was 0.322 for S1, the minimum PCC was 0.998 for both S1 and B1, and the minimum NSE was 0.839 for S1. The data analysis results showed that the model, grid, and parameter settings used in our numerical simulation were appropriate.

**Figure 19:**
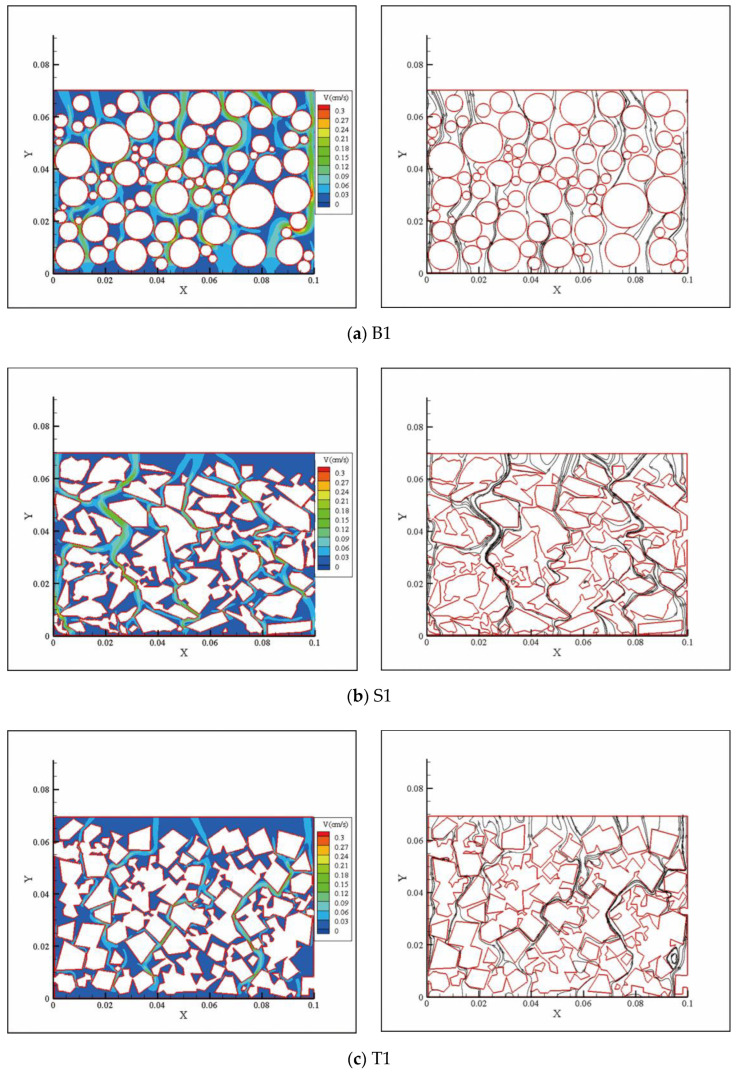
Partial flow field diagrams obtained from the simulation. The left column graphs are the velocity field diagrams with a hydraulic gradient of 0.1, and the right column graphs are the streamline diagrams with a velocity greater than 0.09 cm/s, flowing from the inlet to the outlet with the same hydraulic gradient.

Figure 19 shows the flow fields of the models for the D1 particle size distribution with the 0.1 hydraulic gradient. We found that the flow field differences between the models were similar. Due to the limited space in this paper, we only present the model calculation results for the D1 particle size distribution for illustration purposes. The streamline diagrams with a velocity greater than 0.09 cm/s and running from the water inlet to the water outlet with the 0.1 hydraulic gradient are also shown in the same graph.

The model presented a preferential flow according to the velocity field diagrams in Figure 19. The lighter color bands in the velocity field represent the path of the water particles with a velocity greater than 0.9 cm/s. Under pressure from the bottom to the top, the water did not flow evenly along all waterways but concentrated on a few paths. Macropore flow, bypass flow, and pipe flow existed in the models. These all belonged to typical preferential flow phenomena. As can be seen from the graph, the number and diameter of the preferential flow channels in the sphere pack model were the largest, and those of the octahedron pack model were the smallest. There were more preferential flow channels in the sphere packs from the inlet to the outlet. The preferential flow of the model of the other two shaped packs diverged after encountering the corner, showing a decrease in the flow rate from the macroscopic perspective.

**Figure 20:**
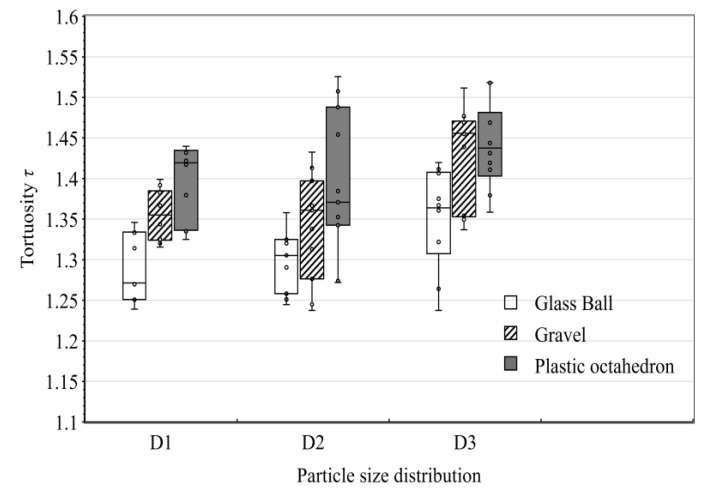
Box plot of the tortuosity of the traces in the flow field from the simulation.

The streamline is a curve that is tangent to the velocity vector at every point in the flow field, while the trace is the curve depicted by the fluid mass as it moves through space. For a constant flow, the streamline and trace coincide. Therefore, as long as the tortuosity of the streamline is calculated, the tortuosity of the trace is obtained. The number of seed points for all simulation results that generated the streamlines was set to 10. The tortuosity (*τ*) is an important parameter that describes the seepage channels. It is defined as the actual length of the seepage channel and the apparent length through the samples; that is, it is the exact length of the water’s motion track in the channel when the permeate water passes through a unit distance of a pack:

(21) $$\tau =\frac{L_{t}}{L_{0}}\;$$

where *L*_t_ is the length of the curved line and *L*_o_ is the length of the line along the direction of the hydraulic gradient. Figure 20 depicts the statistical results of the tortuosity of the traces of all models. According to the box plot, for the same particle size distribution, the *τ* of the ball pack was the smallest and that of the octahedron pack was the largest. The greater the tortuosity, the longer the actual path of water flow, and the macroscopic results were that the cross-section flow of porous media decreased and the permeability decreased over time. The same material pack had the smallest *τ* for D1 and the largest *τ* for D3, which is consistent with the law of hydraulic conductivity. The distribution of *τ* was not uniform because the two-dimensional simulation of the seepage field had limitations. Since a three-dimensional porous media pore network model is too complex in space to mesh appropriately, we only simulated the laboratory test in two dimensions. Although the *τ* in Figure 20 does not represent the actual tortuosity of the water path of porous media in the laboratory test, our two-dimensional simulation was based on the CT images of the packs, and the pore structure in the model was the same as the actual pore structure. The characteristic of the flow field is convincing.

### 3.4. Contributions, Applications, and Limitations

In this study, the influence of particle shape on the pore characteristics and permeability of gravel was studied by combining laboratory tests and simulations. The contributions and applications are as follows:

1. A method for estimating the shape of a gravel pack via manual sampling and measuring the shape factor of gravel with three-dimensional physical meaning was proposed. Previous studies showed that the hydraulic conductivity of the filter material in a natural quarry is higher than that in a blasting quarry under the same conditions [3,22]. However, because the shape of the gravel pack is difficult to quantify through the laboratory test, few studies have considered the particle shape in the design and selection of the filter material. Many dams are built in alpine and canyon areas for higher economic benefits. They use blasted gravel as dam building materials, which is different from the current design specification and does not consider the particle shape. According to our results, for better anti-seepage, the dam building material should preferably be blasted gravel with a large aspect ratio and small roundness. After particle size sieving in the stockyard, gravel of the same particle size group can be sieved again for particle shape using a rectangular or rhombus sieve.
2. A method for measuring the surface area of gravel and its pore network based on SVM segmentation and the reconstruction of CT images was proposed. This method can self-adjust the parameters through deep learning to measure the surface area of particles with different densities and sizes, which is suitable for engineering applications. In addition, it has few requirements in terms of vessel materials and can be coupled with other hydraulic and mechanical tests. This method increases the practicability of the formula for predicting the hydraulic conductivity of gravel using the specific surface area in engineering applications [11,14,15,16,62,63,64,69,70,71]. During a dam’s construction, a laboratory is set up on the site to test the particle size distribution and dry density of the filled part to control the construction quality. As filling uses rolling technology, the gravel may break during the rolling process, which causes the actual particle size distribution and dry density of the filling material to deviate from the design value. Based on the consensus that material with a more significant specific surface area has low permeability [11,14,15,16,62,63,64,69,70,71], the specific surface area can be added for construction control in the filter using our measurement method.

A limitation of this study was that the number of research samples was small due to the heavy workload of manually measuring the gravel shape. A new formula for predicting permeability by considering the gravel shape has not been proposed. In addition, due to the difficulty of meshing a three-dimensional reconstructed model using CT images, this study only calculated the seepage field of four CT slices of each sample from the two–dimensionality. We will conduct further research to expand the total number of measurements and overcome the obstacles of three-dimensional model meshing.

## 4. Conclusions

This study aimed to investigate the influence of gravel’s shape on its pore structure and permeability through CT scanning tests, permeability tests, and simulations to provide suggestions for the design and construction of the earth rock dams’ filter and increase the application of predictive formulas related to the shape factor and surface area of gravel in engineering applications. Some valuable conclusions were as follows:

1. A new method was proposed to estimate the gravel pack shape; this method involved manual sampling and measuring the gravel’s aspect ratio and roundness with three-dimensional physical significance, which is expected to be popularized for the study of the shape of actual gravel packs and their related hydraulic or mechanical properties. One should pay attention to making the gravel pack’s particle size distribution consistent with the sample’s particle size distribution and control the particle size to the centimeter level when using this method.
2. A new method was put forward that uses SVM segmentation and the reconstruction of CT images to measure the surface area of a gravel pack and its pore network. The advantage of the method is that it can be coupled with other hydraulic and mechanical tests and can automatically adjust the parameters according to different testing materials for convenient use in engineering. The specific surface area can be added for construction control of the filter using this method.
3. A gravel pack with a larger aspect ratio and smaller roundness had a larger box dimension associated with its pore–particle interface and a greater specific surface area of the pore network, which meant it had a more complex pore structure. The content of particles less than 5 mm affected the relationship between the shape factor, pore–particle interface, and specific surface area of the pore network. The influence degree of particle shape was dependent upon the content of fine particles.
4. A gravel pack with a larger aspect ratio and smaller roundness had a smaller hydraulic conductivity. This was because the CT scanning results showed that the larger the aspect ratio and the smaller the roundness, the more contact points and contact relationships there were between the particles in a gravel pack with a complex pore structure. This would increase the number, length, and tortuosity of the seepage channels when seepage occurs in such gravel packs in a two-dimensional simulation seepage field.
5. In addition to allowing the particle size distribution and dry density to meet the requirements of the dam design specifications, the filter material should preferentially use blasting gravel with a larger aspect ratio and a smaller roundness for better anti-seepage performance.

## Figures and Tables

**Table 1:** Test sample information.

Test Number	Material	Name	Particle Size Distribution	Porosity
1	Glass ball	B1	D1	P1
2	Gravel	S1		
3	Octahedron	O1		
4	Glass ball	B2	D2	P2
5	Gravel	S2		
6	Octahedron	O2		
7	Glass ball	B3	D3	P3
8	Gravel	S3		
9	Octahedron	O3		

**Table 2:** Mixing proportion of particles.

	Particle Size (mm)	Particle Size Distribution		
		D1	D2	D3
**Proportion (%)**	2~5	3	30	45
	5~10	16	20	30
	10~20	81	50	25

**Table 3:** Parameters of the pack.

Particle Size Distribution	Material	Name	*φ* (%)	*α_p_*	*S_p_*
D1	Glass ball	B1	38.81	1.00	1.000
	Gravel	S1		2.16	0.862
	Plastic octahedron particle	O1		4.67	0.720
D2	Glass ball	B2	32.29	1.00	1.000
	Gravel	S2		2.09	0.865
	Plastic octahedron particle	O2		4.67	0.720
D3	Glass ball	B3	31.22	1.00	1.000
	Gravel	S3		2.06	0.867
	Plastic octahedron particle	O3		4.67	0.720

*α_p_* is the aspect ratio of the particle pack, *S_p_* is the roundness of the particle pack, and *φ* is the porosity of the pack.

**Table 4:** Comparison of the porosity of the model and the pack.

Test Number	*V* (cm^3^)	*φ_sim_* (%)	*φ_Lab_* (%)	*R* (%)
B1	234.46	38.40	38.81	1.05
S1	259.67	37.80		2.59
O1	212.85	38.74		0.19
B2	177.83	32.36	32.29	0.22
S2	179.89	32.74		1.38
O2	184.25	33.53		3.84
B3	176.45	32.11	31.22	2.85
S3	170.61	31.05		0.55
O3	174.20	31.70		1.54

*V* is the volume of the model of the pore network, *φ_sim_* is the porosity of the model of the pore network, and *φ_Lab_* is the porosity of the pack. *R* is the relative error.

**Table 5:** Accuracy analysis of the simulation result.

Test Number	RMSE	PCC	NSE
B1	0.000	1.000	1.000
S1	0.322	0.998	0.839
O1	0.139	1.000	0.998
B2	0.173	0.998	0.980
S2	0.020	1.000	1.000
O2	0.043	1.000	1.000
B3	0.269	1.000	0.974
S3	0.069	1.000	0.999
O3	0.054	1.000	1.000

## Data Availability

Not applicable.
